# Robot and Neuroscience Technology: Computational and Engineering Approaches in Medicine

**DOI:** 10.1155/2016/5204510

**Published:** 2016-07-25

**Authors:** Hiroki Tamura, Shangce Gao, Qixin Cao, Chuntao Leng, Hui Yu, Harold Szu

**Affiliations:** ^1^Department of Environmental Robotics, Faculty of Engineering, University of Miyazaki, 1-1 Gakuen Kibanadai-nishi, Miyazaki-shi 889-2192, Japan; ^2^Department of Intellectual Information Engineering, Faculty of Engineering, University of Toyama, No. 5506, Information Technology Building (G9), Gofuku 3190, Toyama 930-8555, Japan; ^3^Research Institute of Robotics, Shanghai Jiao Tong University, No. 800 Dong Chuan Road, Shanghai 200240, China; ^4^Engineering Training Center, Shanghai Jiao Tong University, No. 800 Dongchuan Road, Minhang District, Shanghai 200240, China; ^5^School of Creative Technologies, University of Portsmouth, Winston Churchill Avenue, Portsmouth PO1 2UP, UK; ^6^Biomedical Engineering, Catholic University of America, Washington, DC 20064, USA

Robot and neuroscience technology based on computational and engineering approaches, which has been successfully applied to a wide variety of fields such as medicine, provides giant opportunities for the advancement of human-computer interface's application to promote the medical research, improve the quality of life, and enhance the patient's safety. Academia generally takes it for granted that the findings in robot and neuroscience have greatly promoted the development of artificial intelligence and consequently developed robotic technology. The research which focuses on interdisciplinary fields includes various areas like artificial intelligence, models, and computational theories of human cognition, perception, and motivation and brain models, artificial neural nets, and neural computing. Thus, more and more attention has been paid to the research progress on computational intelligence and neuroscience and their potential applications in robotics.

The scope of this issue has been restricted to items that are relevant to building theoretical and practical systems including contributions in the area of applicable neural networks theory, supervised and unsupervised learning methods, algorithms, architectures, performance measures, applied statistics, software simulations, hardware implementations, benchmarks, system engineering and integration, and innovative applications. There were a total of 15 manuscripts received, six of which were accepted for publication. The adopted articles embrace a scope that is the representative of computational intelligence and neuroscience technologies, namely, rhythmic oscillations with synaptic learning, human-computer interface for communication, interactive astronaut-robot system, novel abrupt change detection framework, ubiquitous robotic technology's application, and object tracking in robot vision system.

The six papers offer many ideas in robot and neuroscience technology.

This special issue sheds light on bridging the gap between neuroscience, artificial intelligence, and engineering. Editors truly hope this special issue celebrates the communal efforts in this field and becomes the academic citation reference for the researchers of robot and neuroscience technology and of the beneficiaries.

